# Learning challenges in Physical Therapy, Speech-Language-Hearing Sciences, and Occupational Therapy undergraduate programs during the COVID-19 pandemic

**DOI:** 10.1590/2317-1782/20232022025

**Published:** 2023-07-03

**Authors:** Alessandra Giannella Samelli, Carla Gentile Matas, Naomi Kondo Nakagawa, Talita Naiara Rossi da Silva, Silvia Maria Amado João

**Affiliations:** 1 Departamento de Fisioterapia, Fonoaudiologia e Terapia Ocupacional, Faculdade de Medicina - FM, Universidade de São Paulo - USP, São Paulo (SP), Brasil.

**Keywords:** Covid-19, Teaching, Education, Distance, Student, Learning, Covid-19, Docentes, Educação a Distância, Estudante, Ensino

## Abstract

**Purpose:**

COVID-19 posed numerous challenges to educational programs that had to quickly adapt to remote online learning (ROL) to ensure the continuity of health professional training over the pandemic. We aimed to assess the students' and professors' perceptions of the teaching-learning process in the Physical Therapy, Speech-Language-Hearing Sciences, and Occupational Therapy undergraduate programs at a Brazilian public university.

**Methods:**

We used an electronic self-reported questionnaire with multiple-choice questions on a Likert scale ranged 1-5; higher the score, higher the level of agreement/importance/satisfaction.

**Results:**

Most of undergraduate students and teachers had previous experience using information and communication technologies, and 85% stated their preference for in-person learning. Students expressed their appreciation for more active learning methodologies with clear objectives, accessible content, and visualization of abstract concepts. Regarding benefits and barriers, some similar perceptions were observed between students and teachers with ROL favoring time management, benefits in the teaching-learning process, satisfaction and motivation with the course content, and low attendance rates to general academic activities due to absent or poor access to technological resources.

**Conclusion:**

ROL is an alternative learning mode when the in-person classes cannot be carried out, as in the case of the COVID-19 pandemic. ROL is believed to be unfit to replace in-person learning, although it can complement the traditional classroom-based education in a hybrid model, respecting the nature of each program in the field of health that requires in-person practical training.

## RESUMO

**Objetivo:**

O COVID-19 impôs inúmeros desafios aos programas educacionais que tiveram que se adaptar rapidamente ao aprendizado remoto on-line (ARO) para garantir a continuidade da formação dos profissionais de saúde durante a pandemia. O objetivo do estudo foi avaliar a percepção de alunos e professores sobre o processo ensino-aprendizagem dos cursos de graduação em Fisioterapia, Fonoaudiologia e Terapia Ocupacional de uma universidade pública brasileira.

**Métodos:**

Foi utilizado um questionário eletrônico autoaplicável com questões de múltipla escolha em escala Likert de 1 a 5; quanto maior a pontuação, maior o nível de concordância/importância/satisfação.

**Resultados:**

A maioria dos alunos de graduação e professores tinha experiência anterior no uso de tecnologias de informação e comunicação, e 85% afirmaram preferir o ensino presencial. Os alunos expressaram preferência por metodologias de aprendizagem mais ativas, com objetivos claros, conteúdo acessível e visualização de conceitos abstratos. Em relação aos benefícios e barreiras, algumas percepções semelhantes foram observadas entre alunos e professores com ARO favorecendo a gestão do tempo, benefícios no processo ensino-aprendizagem, satisfação e motivação com o conteúdo do curso e baixa frequência às atividades acadêmicas gerais por ausência ou dificuldade de acesso aos recursos tecnológicos.

**Conclusão:**

O ARO pode ser uma modalidade alternativa de aprendizado quando as aulas presenciais não podem ser realizadas, como no caso da pandemia do COVID-19. Porém, o ARO é inadequado para substituir a aprendizagem presencial, embora possa complementar a educação presencial tradicional em um modelo híbrido, respeitando a natureza de cada programa na área da saúde que exige formação prática presencial.

## INTRODUCTION

The advent of COVID-19, in 2020, imposed not only social distancing but also changes in our way of thinking and learning. We do not know yet how long this situation will last, nor its effects on the post-pandemic “new learning environment”. What can educators do to create an adequate learning environment? What is happening with the students’ teaching-learning process? What can educators do to have an adequate working environment? Are educators and students motivated with this remote learning mode?

Educational systems worldwide felt the effects of the COVID-19 pandemic and have been facing unprecedented challenges. A world report pointed out that about 1.4 billion students in more than 156 countries had not been attending school since the beginning of the pandemic^(1)^. In such a scenario, schools and universities have been resorting, with greater or lesser success, to distance learning (DL) and a variety of remote learning (RL) modes to diminish the impacts of the pandemic on the educational routine.

RL follows the same principles of the in-person teaching-learning process. The strategy is similar to DL in the sense that the professor is not in the same environment as the students and that it is developed with the use of technology - which is not the only option in the case of DL. In its turn, DL requires atemporal support from tutors and greater flexibility on the part of the students to organize their study schedule, thus focusing on asynchronous activities and interactions^(2)^.

Classes have been canceled in Brazil as well, which led the Ministry of Education (MEC) to pass a regulation authorizing classes to be given online instead of in-person in the entire federal school system while the COVID-19 pandemic lasts^(3)^.

This necessary transition from in-person to RL, though temporary, caused countless difficulties to educators. They, much like the students, experience the sanitary, social, and economic restrictions and concerns that either appeared in the pandemic or were aggravated by it. In RL, educators must take on new challenges, which include adapting the content, classroom dynamics, lectures, and assessments, ensuring the quality of the learning process while maintaining the students’ interest and commitment^(4)^.

Studies on the learning process showed that the transition from in-person to remote classes requires a gradual and adaptive process for both educators and students. The unexpected fully online learning imposed by the COVID-19 pandemic has inevitable weaknesses, as students feel more distant from the educators and less focused in the online format^(5-7)^.

Countries with advanced technology have e-learning systems. However, this is not the case in many low- and middle-income countries. For instance, a study conducted in Pakistan during the pandemic, while in-person classes were canceled, revealed that the challenges faced by medical school professors and students included a lack of faculty training, little institutional support, Internet connectivity issues, students’ low commitment, online assessment difficulties, and problems understanding the unique dynamics of remote online learning (ROL). All the people involved need to cooperate and participate to meet these challenges^(8)^.

Studies with Brazilian medical school students verified that most of them agree with the implementation of ROL while in-person academic activities remain canceled because of the pandemic - especially those already in the third or fourth year (63.9%) and fifth or sixth year (66.8%). On the other hand, 66.1% of the participants who are in their first or second year and 56.0% of those in the third or fourth year of medical school suggested that the ROL activities be retaken in in-person classes^(9)^.

On the same perspective, less than half (49.2%) of the pharmacy students at a Saudi university had a positive attitude toward the ROL offered during the COVID-19 pandemic, and 34% pointed out barriers to learning in this mode - which suggests the need for training^(10)^.

Given the challenges imposed on education by the COVID-19 pandemic, this study aimed to assess the students’ and professors’ perceptions of the teaching-learning process in the courses taught in ROL mode throughout 2020 in the Physical Therapy, Speech-Language-Hearing Sciences, and Occupational Therapy programs at a Brazilian public university.

## METHODS

### Study population

This study was approved by the local Ethical Committee (CAAE: 40023420.6.0000.0068). The online survey was conducted between November 2020 and January 2021 and sent to all registered students in the first- to fifth-year Physical Therapy and Speech-Language-Hearing and first- to fourth-year Occupational Therapy and professors who teach these specific courses at the Medical School, University of São Paulo, Brazil. Students and professors participated voluntarily in the study, upon agreement with the informed consent form.

### Survey design and implementation

The research began after its project was approved by the institution’s Research Ethics Committee.

Two questionnaires - one for students, the other for professors - were developed based on a previous study^(6)^. The questions were adapted to Brazilian reality and the undergraduate courses assessed.

With these instruments, the students and professors assessed the value of a variety of RL resources and characteristics; curricular structure; communication, economic, academic, and mental health vulnerability; and adjustment to their study or work setting. Other variables, such as access to technology and family and pedagogical issues, were also included. The questions had multiple-choice answers, many of which were on a Likert scale (ranging from 1 to 5) with the highest score as the greatest level of agreement, importance and satisfaction regarding the subject investigated.

The questionnaires were administered online via Google Forms and available between November 2020 and January 2021. The answers were stored automatically in an Excel databank for analysis. Each questionnaire took about 10 minutes to be filled out. The participation was anonymous and optional, unrelated to any assessment of either the student or professor.

### Statistical analyses

The mean values and standard deviations were calculated, as well as the absolute and relative frequencies when appropriate for descriptive data analysis. In the inferential stage, the ANOVA and chi-square tests were used when pertinent, at the 5% significance level.

## RESULTS

Of the 369 enrolled students, 98 (26.6%) answered the questionnaire (41 Physical Therapy, 35 Speech-Language-Hearing, and 22 Occupational Therapy students). As for the term they were attending, 30 were in their first year (30.6%), 19 in the second year (19.4%), 31 in the third year (31.6%), and 19 in either the fourth or fifth year (19.38%). The students’ mean age was 23.3 years (SD: 6.6), and 81.6% were women. Concerning the courses they had taken, 56.3% were predominantly theoretical, 36.5% predominantly theoretical-practical, and 7.3% predominantly practical. Most of the students (60.2%) had previous experience with information and communications technology (ICT).

Of the 36 professors in the Department of Physical Therapy, Speech-Language-Hearing Sciences, and Occupational Therapy, 20 (55.6%) answered the questionnaire (10 Physical Therapy, 5 Speech-Language-Hearing Sciences, and 5 Occupational Therapy professors). Their mean age was 54 years (SD: 7.2), and 90% were females. A total of 60% of these professors had experience with ICT. All of them taught remote online classes during the pandemic and 90% taught classes with synchronous and asynchronous activities, while 10.0% used only synchronous activities. Of the courses taught by the professors, 68.4% were predominantly theoretical-practical and 31.6%, were predominantly theoretical.

### Students’ and professors’ perceptions of the teaching-learning process

The students’ and professors’ preferences regarding the learning mode are described in Table 1.

**Table 1:** Absolute and relative frequencies of students and professors in relation to learning mode preference.

Mode	n (%) students	n (%) professors	Chi-square test p-value
In-person learning	84 (85.7)	17 (85)	0.075
Asynchronous ROL	7 (7.1)	-	
Synchronous ROL	4 (4.1)	-	
Asynchronous and synchronous ROL	3 (3.1)	3 (15)	
Total	98 (100)	20 (100)	

**Caption:** ROL: remote online learning

The scores given by students and professors to ROL variables are described respectively in Tables 2 and 3.

**Table 2:** Descriptive measures (mean and standard deviation) of the scores (Likert scale) given by the students to the remote online learning variables (strategies, characteristics, resources, and motivation)

**Variable**	**Mean (SD)**
Professor’s synchronous classes	3.92 (0.74)
Students’ presentations	3.05 (0.55)
Video presentations	4.04 (0.80)
Case studies	4.03 (0.78)
Group study	3.39 (0.55)
Clear learning objectives	4.53 (0.13)
Accessible learning content	4.79 (0.17)
Visualizing abstract concepts	4.35 (0.10)
Highlights and summaries	4.12 (0.85)
Instructional material and resources	4.29 (0.10)
Technical support	4.10 (0.88)
Questions and exercises	4.21 (0.92)
ROL helps achieve a good final grade	3.36 (0.47)
ROL helps increase previously acquired knowledge	2.99 (0.46)

**Caption:** SD: standard deviation, ROL: remote online learning

**Table 3:** Descriptive measures (mean and standard deviation) of the scores (Likert scale) given by the professors to the remote online learning variables (strategies, characteristics, resources, and motivation)

**Variable**	**Mean (SD)**
The interaction with the students was sufficient for learning in ROL	3.20 (0.12)
The pedagogical strategies and resources you used in ROL are a motivating factor for the students	3.65 (0.21)
The pedagogical strategies and resources you used in ROL are a motivating factor for your work as a professor	3.20 (0.11)
You felt motivated to use ROL in the teaching-learning process	3.40 (0.12)
Teaching your course with ROL was positive in terms of the value you give it	3.50 (0.14)

**Caption:** SD: standard deviation, ROL: remote online learning

Regarding pedagogical strategies and resources, 100% of the professors referred to required reading; 95% to videos; 80% to clinical case discussions; and 45% to recorded classes. Other options were also included by 10% of the participants (peer discussions, questionnaires and exercises, seminars, simulations, problem-based activities, complementary material, and so forth).

The scores given by students and professors to ROL variables regarding benefits and barriers are shown in Table 4.

**Table 4:** Comparison of the means of the scores (Likert scale) given by the students and professors to the remote online learning variables (benefits, barriers)

**Variable**	**Mean (SD) student**	**Mean (SD) professors**	**p-value (ANOVA)**
ROL helps improve professional skills	2.70 (0.34)	3.40 (0.12)	<0.001
ROL helps develop communication skills	2.62 (0.33)	3.30 (0.97)	<0.001
ROL favors time management	3.31 (0.49)	3.25 (0.10)	0.587
ROL has benefits to learning	3.49 (0.67)	3.45 (0.10)	0.791
I am satisfied/motivated with the content of the program in ROL	2.97 (0.38)	2.90 (0.98)	0.589
ROL met my expectations	2.79 (0.36)	3.20 (0.11)	<0.001
In-person learning is an essential component throughout the health training program	4.52 (0.15)	4.65 (0.28)	<0.001
There are barriers to learning when ROL is used	4.47 (0.12)	4.55 (0.25)	0.030
Learning in ROL is better than in the traditional face-to-face education	1.72 (0.21)	2.10 (0.56)	<0.001
Learning in ROL can be combined with face-to-face education after the pandemic	3.30 (0.48)	3.80 (0.15)	<0.001
It was difficult for me to follow (and/or offer -professor) the remote academic activities due to absent or poor access to technological resources	2.11 (0.50)	2.05 (0.10)	0.657
It was difficult for me to follow the remote academic activities because I needed personal academic support (for lack of assistance - professor)	2.26 (0.14)	2.10 (0.76)	0.052
I reckon there has been an overload of contents developed remotely in comparison with in-person classes (student)	3.76 (0.70)	4.45 (0.66)	<0.001
Remote teaching has caused an overload of work (professor)			
Because of the mandatory social distancing and the longer time spent at home, I had to perform household chores or care for relatives, thus limiting the time I had available to carry out remote pedagogical activities	4.18 (0.10)	3.40 (0.12)	<0.001
I am at greater risk (or there is someone in my household at greater risk) of COVID-19	3.38 (0.52)	3.80 (0.22)	<0.001

**Caption:** SD: standard deviation, ROL: remote online learning

## DISCUSSION

This study assessed the students’ and professors’ perceptions of the teaching-learning process in the courses taught in ROL mode throughout 2020 in the Physical Therapy, Speech-Language-Hearing Sciences, and Occupational Therapy programs at a Brazilian public university.

Concerning the teaching mode (Table 1), 85% of the students and professors preferred in-person classes, corroborating the findings of previous studies^(11,12)^. Similar results were reported in another study^(6)^, in which 54.17% of the students preferred face-to-face rather than online learning. The authors highlighted that ROL is a good alternative when in-person classes are canceled, as in the case of the COVID-19 pandemic, but that it cannot replace in-person learning; instead, it must be only a complement to the traditional learning mode. They further emphasized that, in post-COVID-19 times, it would be desirable to offer a hybrid teaching model combining face-to-face and online learning to provide synergic and complementary instruction^(6)^.

The courses taken by the students were predominantly theoretical and theoretical-practical (56.3% and 36.5%, respectively), whereas only 7.3% were predominantly practical. It must be pointed out, though, that most of the students that answered the questionnaire were in the first years of the Physical Therapy, Speech-Language-Hearing Sciences, and Occupational Therapy programs, whose curriculum at such stage offer basic science courses, i.e., predominantly theoretical ones - taught by professors of other areas, such as Biology, Anatomy, Physiology, Psychology, and others. Moreover, the predominantly practical courses taught to students in the last years (internships) were temporarily canceled at the beginning of the pandemic, following the recommendations of the university to meet the social distancing sanitary measures taken during the pandemic.

Authors ^(13)^ emphasize that, as part of the social distancing measures, the professors had to quickly rearrange their classes and other activities, including research and clinical practice, to online platforms. However, they also point out that not everything was feasible, which temporarily decreased the course load of the predominantly practical classes. This is noticed in the results of the present research as well, especially regarding the professors’ answers.

Different from the students’ answers about the courses taken, 68.42% of the courses taught by the professors were predominantly theoretical-practical and 31.58%, predominantly theoretical. It must be pointed out that the professors that answered the questionnaire do not teach the general basic courses offered in the programs’ first years. Rather, they teach each program’s specific required courses. Thus, the courses considered by students and professors in their answers were not necessarily the same.

Most of the students and professors had previous experience using ICT (60.2% and 60%, respectively). All the professors taught ROL classes during the pandemic (90% with synchronous and asynchronous activities and 10% with synchronous activities). Such results corroborate those obtained by previous study^(14)^, who assessed how undergraduate health students perceived the integration of academic teaching with technology. They observed that those students often use digital platforms, which make their learning and professional practice easier, and that they want to increase their knowledge continuously using technology tools while in college. The authors also mentioned that some professors are reluctant to use ICT because of difficulties learning how to handle them; hence, much time and effort would be necessary to adapt to ICT, as they do not master it yet.

Concerning the students’ perception of ROL strategies and resources (Table 2), they preferred the ones with active teaching methodologies (e.g., case studies - mean: 4.03) or more related to practical, know-how activities (e.g., video demonstrations - mean: 4.04; and instructional material and resources - mean: 4.29). Such results corroborate those obtained by another study^(15)^, whose emphasis was on learning based on case studies and problem-solving, thus offering the students an opportunity to work independently.

Some studies (6,16) showed similar results as the present one, highlighting the professors’ lectures as an important teaching strategy (mean: 3.9). They also valued technical support (mean: 4.1), accessible learning content (mean: 4.8), and highlights and summaries (mean: 4.1) as resources to help develop the programs remotely. In the present study, the students also valued clear learning objectives (mean: 4.5) and visualizing abstract concepts (mean: 4.4).

Professors should consider teaching with strategies such as interactive activities involving problem-solving, discussions, and debates during class because they improve the students’ motivation and attitude, creating an active study environment with effective learning. As a result, these educational strategies are considered more effective^(15,16)^, which explains their higher scores in the present study. Active classroom learning helps students acquire higher-order cognitive skills, as they relate new knowledge and skills to what they had previously acquired^(15)^.

Online course developers state that education must be focused on the students‘ needs^(17)^. Hence, they must consider using most of the time more active instructional strategies, in agreement with the results of this study.

The lowest means regarding ROL strategies, resources, characteristics, and motivation (Table 2) referred to increasing knowledge (mean: 2.99) and getting a good final grade (mean: 3.36), considering that students deemed it necessary, after the pandemic, to retake content taught remotely^(9)^. The results of the present study suggest that the students are somewhat reluctant to or suspicious of the teaching-learning process in this mode. It must be taken into account, though, that the pandemic forced the universities to quickly transition from in-person to remote teaching, not allowing the professors the necessary time and training to reorganize their courses. Likewise, the students did not have time to get prepared for remote learning.

The professors gave lower scores to variables related to ROL strategies, characteristics, resources, and motivation (Table 3). None of the means was above 4, and the lowest ones referred to teaching motivated by pedagogical strategies and resources used in ROL and to the interaction with students in this teaching mode. Such aspects corroborate the data obtained by previous study^(18)^, who mentioned that some professors still have difficulties handling new technologies, particularly when it comes to planning videoconferences and using interactive methods. They also reported that, even in well-adjusted programs, clinical case discussions structured for virtual classrooms demand greater interaction effort from the professor and students - which in turn decreases the quantity and quality of classroom discussions.

Likewise, authors^(9)^ highlighted that the professor’s emotional status interferes with their teaching activities. It also affects the students’ learning, motivation to put new technologies into practice, and resistance to innovations. The authors emphasize that medical schools should offer emotional and pedagogical support to their professors and students, considering the challenges posed by the COVID-19 pandemic.

Similar perceptions were observed between students and professors regarding many of the ROL benefits and barriers (Table 4). The ones that stood out were “ROL favors time management” (means: 3.31; 3.25) and “ROL has some benefits” (means: 3.49; 3.45). Concerning time management, our results agree with the ones obtained by other authors^(18)^, who verified that offering asynchronous ROL classes allowed students to organize their studying time and consequently develop time-management skills. Moreover, ROL reduces the time the students take going from one place to another - which, combined with the significant decrease in scheduled classes and the effectiveness of the faster video classes, helped the students find more time to engage in extracurricular activities, such as research and mental and/or physical health self-care^(7,18)^. Also, authors^(7)^ reported that about two-thirds of the students complimented the increased flexibility, indicating it as the best aspect of the curriculum in remote learning.

Furthermore, regarding the perceptions of ROL benefits and barriers (Table 4), the lowest scores (i.e., means close to 2.0) were given to “ROL is better than traditional face-to-face learning” (means: 1.72; 2.10; p<0.001) and “difficulties following (or offering) remote academic activities due to absent or poor access to technological resources” (means: 2.11; 2.05; p=0.657).

Concerning the preference for traditional face-to-face learning, our results corroborate those obtained by previous studies^(6,12)^. This last study verified with a longitudinal assessment of an online program that the students were satisfied with its content and reported that it had many benefits. On the other hand, they emphasized that online learning is not better than traditional face-to-face learning, agreeing, though, that they could be combined in the future. The face-to-face environment favors learning in both lectures and laboratory classes, as the students get personally more involved with the professors and less distracted by the surroundings^(12)^.

As for the second variable with the lowest means, “difficulties following (or offering) remote academic activities due to absent or poor access to technological resources”, the data indicate that few participants faced such difficulties. However, it must be pointed out that the college to which these programs belong, in a joint effort with their students’ councils, lent computers (Chromebooks) and Internet SIM cards to some of the students to make ROL possible during the pandemic. Moreover, aided by the Medical Education Development Center (CEDEM, its Portuguese acronym) and volunteer students, the professors had the opportunity to improve their know-how on the use of online platforms and adjust their teaching objectives and classes to ROL. Hence, there was an evident effort on the part of the college to maintain the quality of the undergraduate programs, which would explain the scores^(18,19)^.

The perceptions regarding the other ROL benefits and barriers (Table 4) were not as balanced between the groups. Rather, there were statistically significant differences, for instance in “ROL helped improve professional skills” (means: 2.70; 3.40); “ROL helped develop communications skills” (means: 2.62; 3.30); “There was a need to perform household chores and assist relatives due to social distancing and longer time at home, thus limiting the time available to carry out remote pedagogical activities” (means: 4.18; 3.40).

Similar results were presented by other authors^(12)^, who point out the distraction caused by the family environment as a barrier to ROL. The same study emphasized some of the professors’ concerns, such as the absence of practical activities and the unproductivity of some students who lacked self-discipline. In another study^(6)^, the students brought up other limitations, such as the absence of clinical practice and in-person communication with the professors and other classmates. Likewise, students in a medical school in California identified the lack of learning clinical skills and the absence of practical laboratory activities as the greatest deficits of the curriculum in remote learning^(7)^.

Meta-analysis studies on interactivity in online programs concluded that increasing the interaction between students and professors can contribute positively to the students’ learning experience^(20)^. They also pointed out that this can be provided with text- or audio-based online discussions (for instance, in videoconferences), increasing the students’ satisfaction^(21)^.

As for the students’ overload observed in this research (Table 4), digital fatigue has been pointed out as a barrier to the students’ involvement and efficient learning in ROL. Hence, a suggestion for future initiatives is the development of more efficient remote learning curricula, in which small group sessions lasting 3 or 4 hours are replaced with shorter modules^(7)^.

The unprecedentedly canceled in-person classes led to the need for developing educational strategies that suited ROL. However, various studies demonstrate that transitioning from face-to-face to online teaching-learning model is not an easy task for either students or professors. Its success requires a gradual and adaptive process^(4,12,22)^.

Besides the difficulties of such a transition, attention must be paid to the nature of the programs and the requirements of professional training - which is necessary for the undergraduate health programs, including Physical Therapy, Speech-Language-Hearing Sciences, and Occupational Therapy. However, conducting them exclusively in a distance or remote learning mode impairs the future professionals’ comprehensive training, as well as the integration between teaching and not only services but especially the community and its health needs^(2,23)^. Therefore, despite the experiences during the pandemic that proved it possible to conduct the programs in RL, it is essential to highlight that it took place as a response to a worldwide sanitary crisis that has been lasting for more than a year. Hence, to adopt new teaching-learning models such as hybrid classes, further studies must be conducted to asses which curricular components are benefitted from online educational strategies, thus ensuring quality training^(4)^.

This study has limited potential for generalization because it was conducted in the Physical Therapy, Speech-Language-Hearing Sciences, and Occupational Therapy programs at a single higher education institution. Also, there may have been volunteer response bias, in which people with stronger opinions present more exacerbated either positive or negative perceptions of the matter at hand. Moreover, to ensure the students’ and professors’ anonymity, no sociodemographic data were surveyed. Hence, the perceptions could not be analyzed in relation to socioeconomic aspects, including age, sex, race, and/or ethnicity. Despite the limitations, this is an unprecedented study, assessing the perceptions of Physical Therapy, Speech-Language-Hearing, and Occupational Therapy students and professors about ROL in the context of the COVID-19 pandemic. Although various studies have been developed, particularly with medical and pharmacy students, no research was found approaching the ROL experiences in the three programs studied in this one.

Concerning the future perspective, it is important to conduct a longitudinal study, following up the students’ and professors’ perceptions at different moments of the courses during the pandemic. A more in-depth analysis of the questionnaire is also necessary regarding the theoretical, theoretical-practical, and practical courses.

Regarding education, the COVID-19 pandemic brought at least one positive aspect, as it stimulated changes: in a short time, educators were led to think, to innovate, to practice, to evaluate and to research to adapt he present and the future to new realities. This milestone will lead to educational institutions to change attitudes towards curricula, teaching, learning and assessment methods, as well as towards approaches to students and teachers, seeking a balance between the old and the new^(24)^. But for this to happen in an organized and systematic way, educational practices and innovations must be registered and evaluated, as they will serve as the basis for shaping the future of education and training of health professionals^(25)^.

## CONCLUSION

Based on the students’ and professors’ perceptions presented in the results of this study, we concluded that ROL is an alternative learning mode when in-person classes cannot be carried out, as in the case of the COVID-19 pandemic. ROL is believed to be unfit to replace in-person learning, although it can complement the traditional classroom-based education in a hybrid model, respecting the nature of each program in the field of health that requires in-person practical training.
